# Internal Structure of the Work–Family Conflict Questionnaire (WFCQ) in Teacher Teleworking

**DOI:** 10.3390/ijerph20020970

**Published:** 2023-01-05

**Authors:** Henry Santa-Cruz-Espinoza, Gina Chávez-Ventura, Julio Domínguez-Vergara, César Merino-Soto

**Affiliations:** 1 Escuela Profesional de Psicología, Universidad Autónoma del Perú, Lima 15842, Peru; 2 Instituto de Investigación en Ciencia y Tecnología, Universidad César Vallejo, Trujillo 13009, Peru; 3 Dirección de Investigación, Universidad Tecnológica del Perú, Lima 15046, Peru; 4 Instituto de Investigación de Psicología, Universidad de San Martín de Porres, Surquillo 15036, Peru

**Keywords:** validity, reliability, psychometric properties, confirmatory factor analysis, factorial invariance

## Abstract

The interference between family and work roles has led to the development of scales for their measurement. However, instrumental studies of work–family conflict have not been conducted in the context of teacher teleworking during the COVID-19 pandemic. For this reason, the objectives of this study were set to obtain evidence of the internal structure and fairness of the Blanch and Aluja Work–Family Conflict Questionnaire, as well as its association with job satisfaction and other sociodemographic variables. A total of 235 Peruvian school teachers between the ages of 24 and 72 years (M = 43.79 and SD = 9.67) responded to the scale using the online form. The analysis employed the non-parametric item response theory modeling (Mokken scaling analysis). The structure of two correlated factors was confirmed: work conflict in the family (WCF) and family conflict in the work (FCW). Both dimensions were invariant with respect to sex group and educational level. The association of both dimensions with job satisfaction was theoretically convergent, and the gender of the teachers slightly moderated this relationship. The reliability was adequate for group research. Finally, the instrument can be useful in the organizational context of teachers who telework.

## 1. Introduction

During the COVID-19 pandemic, teachers at all educational levels worked remotely [1] and had to meet the demands of students, parents, and administrators of their educational institutions. The work tasks were performed at the worker’s personal residence, with a fixed or flexible schedule, which fundamentally defines teleworking [1,2,3]. To do so, they had to adapt quickly to virtual teaching and work [2] while dealing with the uncertainty associated with the pandemic, which had mental health consequences [3,4]. Teleworking implied investing a greater amount of time [2], in addition to simultaneously attending to the needs of their family members who were in quarantine to prevent the spread of COVID-19. This generated greater conflict in the fulfillment of family and work roles, especially for parents of school-age children, since they had to provide support during virtual classes, supervision, and daily care [5]. In view of this, considering that teleworking at home with fixed schedules affects mental health [6], it is assumed that the work–family conflict and vice versa could have been present during the remote work of teachers.

Work–family conflict (WFC) is the interference in the fulfillment of family or work roles due to satisfying the demands of one of them [7]. It affects mental health [6,8] and sleep quality [9]; and it is associated with depression [9,10] and emotional exhaustion [11,12]. In the work setting, it has been related to work overload [4,13], burnout syndrome [14], turnover intention [15], and negativity toward work engagement [16], organizational support [17], workers’ professional development [18], as well as to job and life satisfaction mediated by psychological capital and emotional exhaustion [19]. At the family level, work–family conflict is linked to parental stress [13].

The reported consequences of work–family conflict affect personal, family, and organizational well-being and, during the COVID-19 pandemic, they may have been exacerbated by teleworking, since work and family needs have converged in the same physical environment. During confinement and teleworking due to the pandemic, the workload increased, as well as psychological problems, which resulted in teachers feeling overwhelmed. However, they did not stop fulfilling their teaching duties [4] but, as a result, parenting became more stressful, leading to conflict between work in the family and family at work, which in turn negatively impacted job satisfaction [13].

Work–family conflict interference has been taken two directions: work conflict or interference with the family and family conflict or interference with work. Therefore, both aspects are commonly distinguished in the measurement of work–family conflict. In the availability of several instruments, a study [20] compared the psychometric properties of four Work–Family Conflict Scales (WFCS; [21,22,23,24]) and even though the same conceptual definition was found, there were differences in their operational definition, extension, and research sample, with strengths and limitations. The Carlson scale [21] is multidimensional and includes time-based conflict, tension, and behavior, whereas the other scales are unidimensional.

In the referred research, the Carlson et al. [21] test (18 items) was highlighted due to its relationship with other constructs (affective commitment, job satisfaction, turnover intention, and exhaustion), as well as its good discrimination between high and low levels, except for its behavioral items that do not differentiate those evaluated in the continuous range. Nevertheless, the scale has been used in other studies [25,26,27]. In contrast, the Netemeyer scale [24] (ten items) discriminates against those who experience moderate levels of work–family conflict, but it includes three problematic items. The four scales presented a good fit (χ^2^/df < 3). On the other hand, the Fisher scale [27] is questioned because it includes one item to measure work–family conflict (WFC) and another for family–work conflict (FWC), which affects the obtaining of valid and reliable evidence. In contrast, Grzywacz’s brief scale (six items) [23] did not distinguish between the two types of family–work and work–family conflict, since it is a unidimensional scale.

Other investigations have also been reported, such as that of Kopelman et al. [28], which evaluated the construct validity of three integrated scales measuring work, family, and the role of conflict (WFCS, the Work–Family Conflict Scale). From the item analysis, a structure of three correlated factors was obtained, which was confirmed in another study [29] and discussed in other research where a unidimensional structure was found in a sample of Chinese penitentiary police officers, with the principal components procedure [30] questioned for factor analysis [31]. Another scale, that of behavioral role conflict between work and family [32], adapted to other contexts [33,34], detects, in addition to the perception of the conflict, some behavioral manifestations of it, so its length is 82 items. There is also the “Work-Family Conflict Questionnaire”, by Blanch and Aluja [35], constructed between 2003 and 2004; it has an adequate psychometric report on service workers (administration and management, technical personnel, and education) in the city of Lleida (Spain). In addition, in homogeneous Hungarian social service populations and heterogeneous Hungarian populations in the education, health, IT, office work, and customer service sectors, the findings confirmed the quality of the original instrument [36].

The Work–Family Conflict Questionnaire (WFCQ, Blanch and Aluja [35]) was chosen for this study because it is brief, which is a necessary condition in the organizational setting. The language is Spanish, so its use in the Spanish-speaking context avoids misunderstandings in the translation process of the instrument. The content of the items shows differentiated but internally consistent behaviors. In addition, the target population of the original study consisted of different service occupations, among which teachers were included [35,36]. Its internal structure of two correlated factors obtained in the original study in the Spanish population [35] was replicated in the Hungarian population [36]. For its validity evidence with other variables, its dimensions (work conflict in the family [WCF] and family conflict at work [FCW]) were associated with emotional exhaustion, physical symptoms [35,36], job satisfaction, and personal well-being [35], all with theoretical consistency. These pieces of evidence were in line with those found in other measures of family conflict, such as convergent validity with job stress, interpersonal relationships [26], burnout syndrome, job strain, life satisfaction, and the role conflict [24]. In contrast, the divergent validity of work–family conflict was obtained with career ambition [26].

### 1.1. Theoretical Basis of the WFCQ

Instruments for measuring work–family conflict assume various theories [37,38,39,40]. The theoretical perspective of the WFCQ recognizes the importance of social support in lessening the impact of work/family conflict stressors [41]. In addition, work/family stressors affect the family/work domains, with the presence of distress and depression [42]. However, five modalities explain the relationship between family and work [29], including (a) the spillover model, whereby attitudes and affects experienced in one domain (work/family) are transferred to the other domain; (b) the compensation model, which implies a balance or trade-off between the domains due to opposing experiences in them; (c) the segmentation model, which considers that functioning in the domains is independent of each other; (d) the instrumental theory, which posits that one domain provides resources to the other domain; and (e) conflict theory, whereby success in one domain implies sacrifices in the other domain.

To test the theoretical assumptions, studies have been developed to obtain the psychometric properties of the instruments. In the case of the WFCQ, the research is still insufficient [35,36]; however, with what has been reported, we intend to overcome, in this research, the following gaps: (a) the research population consisted of on-site workers and no psychometric reports were found in teachers who perform telework, due to the COVID-19 pandemic. (b) The antecedents have been carried out only in Spanish [35] and Hungarian [36] samples, and no reports have been found in Latin American countries. (c) The replicability of the method used in the original study [35] has focused exclusively on obtaining evidence of validity in relation to other variables [36] and on internal structure, by means of confirmatory factor analysis and internal consistency. Moreover, measurement invariance was only carried out in the original study considering gender and not the teaching level of the teachers in the sample. (d) The constructs defined by the instrument are not used in Latin America, in spite of the Spanish language and the lack of new empirical evidence to confirm the theoretical structure of the questionnaire.

Since it is necessary to have a measure of work–family conflict in this new context, we intend to contribute to the psychometric evidence of the scale in an unprecedented context, where teachers of regular basic and higher education carry out their work in the virtual modality during confinement due to the COVID-19 pandemic. This will enable the proper interpretation of their scores and the understanding of their constructs from an emic perspective, that is, not only contextualized to the study sample (i.e., Peruvians) but also to their potential cross-cultural invariance. Therefore, the aim is to answer the following question: What is the evidence of internal structure and fairness of the Blanch and Aluja Work–Family Conflict Questionnaire for Peruvian teachers who telework? as well as to fulfill the objectives of the study. The objectives of this study are to obtain evidence of the internal structure and equity of the Blanch and Aluja Work–Family Conflict Questionnaire, according to sex and level of education, as well as to determine the association of the dimensions of work–family conflict with job satisfaction and other sociodemographic variables in Peruvian teachers of regular elementary and higher education.

### 1.2. Conceptualization and Hypothesis Formulation

The evidence of two different and interrelated areas of conflict (work/family) finds support in studies [25,42]. In addition, the findings in the Spanish [35] and Hungarian [36] samples, reported for the WFCQ scale a structure of two factors that were correlated and invariant according to gender in Spanish workers [35]. In this regard, it is necessary to point out that discrepant findings have been found on different scales of the work–family conflict. In a study it was revealed that the scale is not equivalent according to gender at the level of factor loadings in German employees [26]; however, it is possible that the differences could be attributed to the cultural context because collectivism moderates the relationships between the work–family conflict, and satisfaction [43] and gender roles are shaped by culture. Therefore, the following hypotheses are proposed.

**H_1_:**  *The Work–Family Conflict Questionnaire has a structure of two related factors, composed of work–family conflict and family–work conflict*.

**H_2_:**  *The Work–Family Conflict Questionnaire has a gender-invariant structure.*

Regarding convergent validity, the WFCQ could be related to some sociodemographic variables. There are discrepancies regarding gender, which indicate its association with work–family conflict [44] or its independence [45]; however, the differences may be attributable to the cultural context. Therefore, taking into account that due to machismo in Peru [46], the greatest responsibility for the family, children’s education, and housework falls on women; this would require their greater dedication to the home, even if they work; and, therefore, women could be prone to conflicts at work due to family interference or conflicts in the family, due to work interference. This was reported in a previous study of working women, in whom it was found that COVID-19 had a negative impact on work–family conflict [47]. There is evidence that work–family conflict is directly related to the number of children [16,17] and dependence on older relatives [16,47]. In addition, the inflexible hours of teaching work and the remote modality of work are added, where work and family demands converge, which could be counterproductive [48] since it is not possible to reconcile personal and professional interests [49]. In this sense, the evidence suggests the following hypotheses.

**H_3_:**  *Conflict in the family due to work differs according to gender.*

**H_4_:**  *The WFCQ shows convergent validity through the positive relationship of its factors (WFC and FWC) with the number of sons/daughters.*

**H_5_:**  *The WFCQ, through its factors, is positively related to responsibility for the care of a family member.*

**H_6_:**  *The WFC factor is positively related to the performance of housework in female teachers.*

In relation to accompanying the academic work of children, studies have shown that parents with greater academic preparation have a positive influence on their children’s mathematics performance [50] and that the parents’ level of education influences the students’ professional aspirations [51]. Therefore, it would be expected that an educator would give importance to academically supporting the work of children, especially those who have not yet achieved their own regulation and are dependent on their parents; for this reason, it is proposed as a hypothesis.

**H_7_:**  *The WFCQ presents convergent validity through the positive relationship of its factors with support for the son’s/daughter’s academic work.*

In relation to age, a study on nurses reported a negative relationship with work–family conflict [52]. It could be expected that the more mature the person is, the more experience and balance he/she will have in managing conflicts in different areas of life. In the workplace, this would correspond to the theory of justice, which explains that people with more experience and training have more resources that allow them to reduce work–family conflict [38]. This approach finds empirical support in a study conducted on Filipino nurses, where a negative relationship, although weak, was found between years of professional experience and work–family conflict [52]. Therefore, the following hypotheses are proposed.

**H_8_:**  *Age is negatively related to work–family conflict.*

**H_9_:**  *Years of teaching experience have a negative relationship with WFC.*

Finally, previous studies have obtained convergent validity of the work–family conflict scales with measures of job satisfaction and found a negative relationship [17,52,53,54]. In the face of disagreements, especially occurring at work due to family interference and work interference in the family environment, it would be expected that the worker would find greater displeasure or dissatisfaction with the job. For this reason, the following hypothesis has been put forward.

**H_10_:**  *WFCQ dimensions are negatively associated with job satisfaction.*

## 2. Materials and Methods

### 2.1. Design

The study has a cross-sectional design, focused on obtaining psychometric evidence of the Work–Family Conflict Scale [35].

### 2.2. Participants

The sample consisted of 235 Peruvian teachers from the three levels of education (pre-school, primary, and secondary), who performed their duties by teleworking, while they were in quarantine to avoid COVID-19 infection. Participants ranged in age from 24 to 72 years (M = 43.79 and SD = 9.67), and were mostly female, married, or cohabiting (Table 1). Participants’ number of sons/daughters ranged from 0 to 5 (M = 1.6, Md = 2, and SD = 1.3) and average ages ranged from 10.8 to 45.8 years (M = 11.8, Md = 10.8, and SD = 9.6). In addition, teachers had between 0 and 49 years of professional experience (M = 16.4, Md = 16, and SD = 9.6).

### 2.3. Instrument

*Work–family Conflict Questionnaire* ([WFCQ]; Blanch and Aluja, [35]). It measures work–family interference through two dimensions: work–family conflict (WFC) and family conflict at work (FWC). Its format is a 7-point Likert scale (strongly disagree, quite disagree, slightly disagree, neither agree nor disagree, slightly agree, quite agree, and strongly agree). Psychometric evidence based on the internal structure was found in two samples of Spaniards [35] and two samples of Hungarian workers, where the obtained structure was that of two correlated factors and an acceptable internal consistency.

*Single-item of job satisfaction* [55]. It was measured with a single item (“Taking everything into consideration, to what degree do you feel satisfied about your job, as a whole”), a type of measure that has proven to be adequate for overall satisfaction and sufficient for surveys and applied research [56]. It is scaled into 7 response options (very dissatisfied, dissatisfied, slightly dissatisfied, neither one nor the other, slightly satisfied, satisfied, and very satisfied). Its translation was performed with a focus on neutral Spanish (i.e., absence of regionalisms) by the first author, and was corroborated by the rest of the co-authors; it was then sampled for comprehension by 3 working adults, obtaining acceptability of the clarity of its content. The validity study obtained high correlations with concurrent multi-item measures of job satisfaction, convergent measures of job support, divergent measures of stress and negativity, and associations with health [55]. In Mexico, its association with occupational stressors was theoretically consistent [57].

*Work–family variables.* Several questions were created to assess some characteristics that linked the work and family of the participants, and to include them in the evaluation of the evidence of association with other variables.

Responsibility for the care of family members: Do you have a family member at home who requires special care? The response options were yes/no.

Participation in housework: Do you take care of housework in your home? The response options were totally, partially, and I do not take care of it.

Accompaniment in children’s homework: Are you in charge of accompanying your children’s school and/or academic work? The response options were totally, partially, I do not take care of it, and I do not have children.

Work experience: How many years of teaching experience do you have? The response was the number of years reported by the participant. 

### 2.4. Procedure

Obtaining informed consent and psychometric assessment was performed using Google forms, through social networks, and the WhatsApp messaging application, due to quarantine and social distancing measures to prevent the spread of COVID-19. The sampling was non-probabilistic of an intentional type. Prior to data collection, informed consent was requested. For this purpose, the research team was introduced, the objective of the study was explained, as well as the estimated time for data collection and the anonymous and voluntary nature of participation. The researchers’ e-mail addresses were also provided to answer any queries related to the study.

*Analysis.* In the first place, detection of possible patterns suggestive of potential inattentive responses or insufficient effort/careless responses was made in a nonparametric item response theory framework [58,59], so that a methodological line with the main analyses was maintained. The Gplus index was used, based on the Polytomous Guttman Errors [59,60], and interpreted as a statistical person-fit. Detection was performed on GPlus scores beyond their interquartile range. The R Mokken program [61] was used. 

Secondly, item analyses were performed with descriptive statistics and response frequency analysis. The R Companion [62] program was implemented. Third, the internal structure was examined. The sample size of the present study imposes some limitations to properly estimating the approaches, such as structural equations modeling or item response theory (SEM and IRT, respectively; [63]), so psychometric parameters were obtained by nonparametric item response theory modeling, including the DIF test [64]. In this way, the Mokken scaling analysis framework (MSA [65]) was used to evaluate four basic properties [63,66,67]: (a) dimensionality (through automated item selection procedure, AISP [61]); (b) local independence (the absence of local dependence or covariation among items once the variance of the score is removed), through the coefficient W(1) [68]; (c) the monotonic homogeneity property, through the number of violations identified by the weighted criterion crit < 40 [61]; and (d) the scalability of items and scores (coefficient H > 0.30; [61]). Within the MSA framework, reliability was assessed with the MS coefficient for ordinal variables [69] using the R Mokken program [61].

Taking into account the observed score of each dimension, the partial gamma coefficient (γp; [70]) was used to evaluate the violation of local independence and differential item functioning (DIF; [71,72,73,74,75]). To reduce the false discovery rate (FDR) due to multiple testing, the Benjamini–Hochberg procedure [76] was used. The magnitude of LD and DIF was evaluated based on the recommendations of Cox et al. [77]. Regarding *p*-value size, weak (*p* > 0.01), moderate (*p* > 0.001), and strong (*p* < 0.001). Regarding γp size, there do not appear to be accepted and agreed-upon levels of effect size; for example, there is the proposal by Bjorner et al. ([71]; null or trivial: < |0.21|, mild or moderate: > |0.21|, and between moderate and large: > |0.31|), Schnohr et al. ([75]; weak < 0.15, moderate: ≥0.15, and strong: ≥0.29), and Healey [78] (weak: ≤0.30, moderate: >0.30, and strong: >0.60). For this reason, a heuristic conclusion was made according to these classification proposals. Additionally, decisions to support the use of γp were based on the relevant literature [67,79]. The analyses were performed with the R iarm program [80].

The association with external variables was performed with nonparametric association coefficients, interpreted as effect size (glass rank biserial correlation, Spearman correlation, and epsilon squared; [81]) of the relationship between WFCQ scores and other variables. All association coefficients were supplemented with 95% confidence intervals and were generated using 1000 bootstrap samples. The R Companion program [62] and bootcorci [82] were implemented. The magnitude of these associations was framed at the following levels [83]: <0.10 (trivial), ≥0.10 (small), ≥0.20 (moderate or typical), and ≥0.30 (large). Because gender can be a factor that interacts with caregiving activities and the solution of household chores, we obtained, independently for men and women, the relationship of the WFC/FWC with responsibility for the care of a family member and with support for the academic work of the children. Subsequently, the magnitude of the correlations of the two groups was compared using Cohen’s q.

To improve the accuracy of the WFC and FWC scores for the presentation of their latent variables [84] and use them in the association with external variables, maximum likelihood scores (sML) were obtained for each participant, generated with Ramsay curves [85,86], a procedure derived from nonparametric item response theory. This method finds weights of the item-observed score relationship by means of smoothed kernel curves regression and estimates sML scores regardless of the distribution of the latent variable. The R KernSmoothIRT package [87] was used.

## 3. Results

### 3.1. Potential Inattentive Responses

None of the participants were identified as generating careless responses (0.0%), so all of the participants were included in subsequent analyses.

### 3.2. Item Analysis

In Table 2 (response frequency heading), unimodality was observed in option 5 of the items theoretically belonging to the WFC scale (items 1 to 4), while the items of the FWC scale (items 5 to 7) unimodality occurred in option 1. This was consistent with the distribution statistics because, in general, the statistics showed clear differences based on the content of the theoretical dimensions and the WFC items indicated greater intensity (M_items_ > 4.00), such as negative asymmetric distribution and positive kurtosis. In contrast, FWC items were less intense (M_items_ < 3.00).

Mardia tests for multivariate normality indicated that this distribution does not hold for the WFC (skewness test = 156.49, *p* < 0.001 and kurtosis test = 9.43, *p* < 0.001) and FWC (skewness test = 82.66, *p* < 0.001 and kurtosis test = 4.28, *p* < 0.001) items. Univariately, for each item, this was also found using the Anderson–Darling test (AD tests > 8.00, all at *p* < 0.001).

### 3.3. Mokken Scaling Analysis

*Unidimensionality.* The AISP procedure indicated two groups of items: one with the minimum scalability at a high level (Hi ≥ 0.50) and the other group below this level (Table 3). These two groups corresponded exactly with the two theoretical dimensions of the instrument; therefore, the dimensionality of the WFCQ in the present sample is defined by the two theoretical dimensions of the instrument, but with different levels of scaling. This finding gives empirical support for the proposed research hypothesis (H_1_).

*Scalability.* At the item level (Table 3), scalability coefficients Hi were strong (>0.50; 62) for the WFC scale (Hi between 0.65 and 0.77), and moderate (between 0.40 and 0.50; 62) for the FWC scale (Hi between 0.42 and 0.44). This same pattern was repeated for the global scalability of WFC (H = 0.73, 95% CI = 0.66, 0.79), and FWC (H = 0.43, 95% CI = 0.33, 0.52).

*Monotonic homogeneity.* No item presented any level of violation of monotonic homogeneity (Table 3, heading Crit MHM). Therefore, all items in their respective dimensions complied with this property.

*Local dependence.* For the FWC scale, W (1) could not be obtained, probably due to the low prevalence of responses in some of the options (see response frequency analysis, Table 2). In WFC, W (1) ranged from 0.05 to 4.45, and none were identified as moderate or strong local dependence. However, using partial gamma coefficients, between items 1-4, 3-4, and 3-2 (Table 4), strong evidence of local dependence (LD; *p* < 0.001) was found; the magnitude of this possible local dependence tended to vary between weak and strong (according to their confidence intervals).

*Differential item functioning (DIF).* In the three classificatory schemes of DIF magnitude [68,72,75], none of the items showed differential functioning in the gender and educational level group (Table 5). Regarding the level of evidence for the existence of DIF, all *p*-values were weak (*p* > 0.01).

*Reliability.* The reliability of CTF scores was MS-rho = 0.90 (alpha = 0.90) and CFT scores was MS-rho = 0.67 (alpha = 0.66).

### 3.4. Association with Other Variables

In Table 6, the monotonic association with years of work experience (H_9_) and age (H_8_) were statistically significant and predominant at the moderate level, except for years of teaching experience with FWC. Therefore, the hypotheses formulated are accepted. The association between strong job satisfaction in WFC and moderate job satisfaction in FWC provides evidence in favor of the proposed hypothesis (H_10_). Null monotonic associations were found between sML, CTF, and CFT scores with housework (except FWC), responsibility in caring for a family member (H_5_), accompanying children to academic tasks (H_7_), except in the WFC of women, the number of children (H_4_), and gender only in WFC (H_3_); so, the referred hypotheses were rejected.

The relationship between gender and sML of WFC and FWC was further investigated, taking into account the possible moderation of gender, by calculating Spearman correlations separately. Regarding housework, in WFC, the association in men was rs = −0.12 (*p* > 0.10, 95% CI = −0.31, 0.09) and in women rs = 0.05 (*p* > 0.10, 95% CI = −0.11, 0.21); the difference between both correlations can be considered small (q = 0.17; Cohen, 1988, *p* = 0.109). In FWC, in men the association was rs = −0.27 (*p* < 0.05, 95% CI = −0.47, −0.01), while in women it was rs = −0.16 (*p* > 0.05, 95% CI = −0.32, 0.00); the difference between them can be considered trivial (q = 0.05). The finding permits the acceptance of the hypothesis (H_6_), but is circumscribed to the FWC dimension. In both coefficients independently calculated for males and females, there are no apparent statistically significant differences due to the overlapping of their confidence intervals.

Regarding accompaniment in children’s academic work, with WFC, in men the association was rs = 0.08 (*p* > 0.10, 95% CI = −0.13, 0.29), while in women it was rs = −0.21 (*p* > 0.01, 95% CI = −0.36, −0.03); the difference between both coefficients may be approximately moderate (q = 0.29). These results partially support the study hypothesis (H_7_). In contrast, in FWC, in men it was rs = −0.01 (*p* > 0.10, 95% CI = −0.24, 0.24) and in women rs = −0.16 (*p* = 0.05, 95% CI = −0.31, −0.00); the difference between both coefficients can be considered small (q = 0.15).

## 4. Discussion

In the field of work–family conflict measurement, different instruments have been reported, with strengths and weaknesses, for specific objectives of use. However, no psychometric studies of the construct have been found in the telework setting during the COVID-19 pandemic. Therefore, it was justified to find evidence of internal validity of the Blanch and Aluja Work–Family Conflict Questionnaire [35] in Peruvian teachers characterized by the inflexibility of their schedules. The instrument was selected because its design included teachers within the target population; it was developed in Spanish and collects different behavioral samples in a reduced number of items, which makes its application in the organizational field feasible and the psychometric findings are comparable in the Spanish [35] and Hungarian [36] samples. In addition, the Latin American context is a new setting for the study of the instrument; the evidence in relation to other variables and measurement invariance needs to be extended with other variables, which have been included in this study.

The present study confirms the structure of the WFCQ in two correlated factors: work conflict in the family (WFC) and family conflict at work (FWC), obtained in Spanish workers [35] and Hungarians [36], which empirically supports the theoretical basis and the first hypothesis (H_1_), and the bifactor structure and the presence of a general factor [36] is questioned. In addition, the strength of the model is based on the use of various methods for this purpose. The strong scalability of the WFC and moderate scalability of the FWC scale and its items indicated their monotonic homogeneity, so all of them were retained. On the other hand, since no differential functioning of the items was found, according to gender and level of education, the fairness of the instrument is assumed, the aspect that allows accepting the proposed hypothesis (H_2_) and that coincides with the original study, which considered gender [35]. However, the findings differ from a study on German employees, where the same construct was measured but with a different instrument, and it was found that the work–family conflict scale is not equivalent at the level of factor loadings [26]. This absence of metric invariance may be idiosyncratic to the sample.

The reliability obtained was acceptable for FWC and good for WFC, which implies that the WFC has comparatively greater reproducibility between scores than the FWC. However, FWC scores are still usable. Taken together, both scores may be viable for group description. The finding coincides with that found in the investigation of the original test [35], where reliability was acceptable for CFT, especially in women, and good for WFC, in the samples of Spanish workers. Additionally, the results are similar to those reported in the Hungarian study [36], where the internal consistency with the omega coefficient is acceptable for FWC and good for WFC.

The association between sociodemographic external variables and WFCQ scores generally generated different levels of associative strength. The interference of the family with work and of work with the family decreases as the teachers’ age increases, a finding that makes it possible to accept the H_8_. This is consistent with the findings of a study on nurses, where a negative relationship between age and work–family conflict was reported [51]. Maturity and experience gained over the years seem to favor the handling of conflictive situations or prevent them from being generated. The same occurs at the professional level, since years of teaching practice are negatively related to work interference in the family; therefore, evidence is found in favor of the acceptance of H_9_.

None of the work–family conflict factors were related to the number of children of the participants, which disagrees with previous studies [16,17]; however, the age of the children could be a confounding variable, because, in a previous study, work–family conflict was positively associated with having school-age children [5]. In this regard, in this study, the average ages of the sons/daughters were between the stages of puberty, adolescence, and adulthood; therefore, possibly because they were no longer dependent on their parents, the number of sons/daughters is not associated with the work–family conflict, so H_4_ is rejected in mathematics [48].

Responsibility for the care of a family member was not related to WFC or FWC, so the hypothesis formulated (H_5_) is rejected, despite the studies that do find a relationship, referring to the presence of dependent older adults [16,24] or school-age children [5], especially those under 12 years of age [88]. It is possible that the teachers participating in this study may have family/social support (a caregiver) to take care of dependents at home while teleworking. However, further studies are required to confirm or debate this assumption. In addition, as already mentioned, the average age of the sons/daughters placed them in stages characterized by greater self-regulation; therefore, it could be expected that those who had young children at home would have been supported by their older children.

Regarding gender, differences were obtained in the FWC, which is evidence in favor of H_3_, a finding that coincides with reports of greater work–family conflict in women than in men [44,85]. In addition, in both men and women, there is a relationship between FWC and the performance of household chores, so the research hypothesis (H_6_) is accepted. In this regard, it is possible that the Peruvian context, being traditionalist and chauvinistic [46], models gender roles in which the woman is in charge of the children’s education, so that accompaniment in academic activities is associated with greater conflict at work. This does not occur in domestic tasks, which apparently is being assumed by both sexes, and is associated with conflict at work, which possibly reflects a tendency toward equity of functions related to household activities, as women participate in the labor market.

The association between WFC and job satisfaction was theoretically convergent, because both experiences are restricted to the work environment and, therefore, the relationship should be comparatively stronger than the association between FWC and job satisfaction. In addition, a greater work conflict in the family could be explained in terms of the telework that characterized the study participants who were in quarantine due to COVID-19, in contrast to the antecedent [35], where a relationship of moderate magnitude was obtained in the relationship between FWC and job satisfaction, specifically in men. Although in the present study the difference in the relationship between WFC/FWC with job satisfaction was detected as small, this finding points to a difference that converges theoretically [17,52,53,54], so the hypothesis stated (H_10_) is accepted.

One limitation is having worked with a small sample of teachers, selected on a non-probabilistic basis and evaluated through online forms, which affects the external validity of the study. However, the study with Hungarians worked with the same type of sampling, in homogeneous and heterogeneous occupational groups, and the data collection procedure was also through the Internet [36], whereas the study with a homogeneous Spanish sample was applied in person and did not report the type of sampling used [35]. Despite this, the results shown are in line with those obtained in the original research in Spain (two-factor correlated structure, convergent relationship with job satisfaction, factorial invariance according to sex, and acceptable reliability) and in Hungary (two-factor correlated structure and acceptable reliability); therefore, it could be possible that the findings are reproducible and that the construct is potentially generalizable. Therefore, further research will be needed to confirm the findings and contribute to increasing the psychometric evidence for the use of the WFCQ in teleworking. Additionally, finding factorial invariance in professionals from different Spanish-speaking countries could be a relevant contribution to the internal structure of the test.

Another limitation is that since it is a self-report instrument, it could be questioned for possible response bias and social desirability [89], especially if respondents are asked to reveal their identity. Although there is evidence that indicates similar results in anonymous and non-anonymous depression evaluations [90], it could be expected that in organizational contexts the results may be different due to social desirability. In the present study, this was controlled with an anonymous and voluntary evaluation; however, future research could relate the WFCQ to some objective measure, such as observational and psychophysiological recordings. In this way, the evidence of convergent validity of the instrument would be increased, since the existing ones have related the WFCQ to other self-report measures [35].

The nature of the cross-sectional study is another limitation, because the intensity of the conflict may vary over time, which could affect the findings obtained. A longitudinal study would have made it possible to systematize the impact of the change from face-to-face to virtual work on work–family conflict, its negative consequences as possible advantages [91], and its psychometric evidence. Therefore, further studies could consider conducting a longitudinal study and thereby help to understand the changes attributed to maturation and the nature of the construct.

On a practical level, an instrument is available to measure work–family conflict in the population of professionals who contribute to the education of future generations, from childhood to adulthood. In addition, the study responds to a new work scenario—teleworking during the pandemic—so it was necessary to investigate the internal structure of the WFCQ designed for face-to-face work. The detection of CTF and CFT is relevant because it has been linked to other variables that have a negative impact on well-being and could undermine the occupational health of workers. Therefore, the use of the WFCQ as a diagnostic tool for teachers can be used in the Peruvian context, will guide the design of prevention programs in organizational settings, and will enable the development of future research, especially in Latin American countries.

## 5. Conclusions

The research confirms that the WFCQ presents a multidimensional structure of two correlated factors (work conflict in the family and family conflict at work), in Peruvian teachers who performed telework during the COVID-19 pandemic. This structure remains invariant according to gender and the educational level at which teaching is practiced. Likewise, convergent evidence was obtained by verifying the negative correlation between the two dimensions of the WFCQ (especially the CTF) with job satisfaction, age, and years of professional experience. It is, therefore, concluded that the WFCQ is an instrument with adequate psychometric properties and is promising for use in research with teachers. In addition, it can be useful for decision-making on interventions related to occupational health and the restructuring of psychosocial factors at work.

## Figures and Tables

**Table 1:** Distribution of participants by sociodemographic variables.

	*n*	%		*n*	%
Gender			Mode (prior to quarantine)		
Male	83	35.3	Face-to-face	214	91.1
Female	152	64.7	Remote	1	0.4
			Both	20	8.5
Level of teaching practice					
Preschool	18	7.7	Dependent family member		
Primary school	73	31.1	Yes	78	33.2
Secondary school	109	46.4	No	157	66.8
Technical superior	34	14.5			
Higher education	1	0.4	In charge of housework		
			Totally	95	40.4
Marital status			Partially	131	55.7
Single	72	30.6	Not in charge	9	3.8
Live-in partner	30	12.8			
Married	122	51.9	Accompanies sons’/daughters’ academic work		
Divorced	7	3.0	Totally	58	24.7
Widow	4	1.7	Partially	81	34.5
			Not in charge	41	17.4
No. of sons/daughters			No children	54	23.0
0	57	24.3	Missing	1	0.4
1	43	18.3			
2	71	30.2			
3	42	17.9			
4	14	6.0			
5	5	2.1			
Missing	3	1.3			

**Table 2:** Item analysis: descriptive and frequency for items.

	Response Frequency							M	SD	Sk	Ku	AD
Options	1	2	3	4	5	6	7					
Items												
ctf1	18	16	28	21	84	44	24	4.55	1.67	−0.62	0.43	8.94
ctf2	13	15	23	21	77	51	35	4.81	1.65	−0.71	0.22	8.51
ctf3	17	21	24	26	73	48	26	4.55	1.71	−0.56	0.59	7.78
ctf4	20	28	27	20	78	46	16	4.31	1.73	−0.48	0.85	9.20
ctf5	71	50	40	38	26	5	5	2.71	1.57	0.63	−0.44	8.975
ctf6	73	34	22	79	19	6	2	2.84	1.54	0.19	−1.07	13.01
ctf7	92	49	36	43	10	4	1	2.34	1.38	0.73	−0.35	13.45

Note: Sk is the skewness coefficient, Ku is the kurtosis coefficient, and AD is the Anderson–Darling normality test.

**Table 3:** Mokken analysis scaling: dimensionality, scalability, and monotonic homogeneity.

Items	AISP		Hi	Crit (MHM)	rit
	0.50	0.60			
WFC					
ctf1	1	1	0.65	0	0.81
ctf2	1	1	0.76	0	0.90
ctf3	1	1	0.77	0	0.92
ctf4	1	1	0.73	0	0.88
FWC					
ctf5	0	0	0.44	0	0.80
ctf6	0	0	0.42	0	0.77
ctf7	0	0	0.42	0	0.74

Note: WFC is work–family conflict, FWC is family–work conflict, AISP is the automated item selection procedure, Hi is the item scalability coefficient in the score, Crit (MHM) is the weighted criterion for the monotonic homogeneity model, and rit is the item–test linear correlation.

**Table 4:** Evaluation of local dependence, with partial gamma coefficients.

	Assessed Item	Gp	LI	UL	padj.BH			Gp	LI	UL	padj.BH
ctf1	ctf2	−0.12	−0.41	0.16	1.00	ctf2	ctf1	0.23	−0.04	0.50	1.00
ctf1	ctf3	−0.44	−0.66	−0.22	0.00 **	ctf3	ctf1	0.06	−0.20	0.33	1.00
ctf1	ctf4	−0.35	−0.59	−0.11	0.04 *	ctf3	ctf2	0.47	0.25	0.69	0.00 ***
ctf2	ctf3	0.32	0.08	0.56	0.09	ctf4	ctf1	−0.21	−0.47	0.04	1.00
ctf2	ctf4	0.00	−0.25	0.26	1.00	ctf4	ctf2	−0.15	−0.44	0.13	1.00
ctf3	ctf4	0.48	0.26	0.69	0.00 ***	ctf4	ctf3	0.25	−0.01	0.53	0.77

Note: Gp is the partial gamma coefficient, * *p* > 0.01 (weak evidence), ** *p* > 0.001 (moderated evidence), and *** *p* < 0.001 (strong evidence).

**Table 5:** Differential item functioning (DIF), with partial gamma coefficients.

	Gender			Level		
	Gamap	IC 95%		Gamap	IC 95%	
		LI	UL		LI	UL
WFC						
ctf1	0.10	−0.21	0.43	0.13	−0.13	0.39
ctf2	−0.01	−0.35	0.31	−0.11	−0.40	0.17
ctf3	0.08	−0.24	0.42	0.01	−0.28	0.31
ctf4	−0.20	−0.53	0.12	−0.09	−0.37	0.18
FWC						
ctf5	−0.06	−0.32	0.18	−0.08	−0.29	0.13
ctf6	0.00	−0.25	0.27	−0.00	−0.23	0.21
ctf7	−0.11	−0.38	0.15	0.09	−0.11	0.30

Note: WFC is the work–family conflict score, FWC is the family–work conflict score, gamap is the partial gamma coefficient, and LI and UL are low interval, and upper interval. Teaching level: initial, primary, secondary, and higher.

**Table 6:** Association with other variables.

	**WFC**	**FWC**
Association between WFC and FWC scores		
WFC	-	
FWC	0.29 ^a^	-
	(0.16, 0.41)	
Association between WFC and FWC with external variables		
	**WFC**	**FWC**
Gender	−0.09 ^b^	0.19 ^b,^ *
(IC 95%)	(−0.26, 0.07)	(0.03, 0.33)
Age	−0.26 ^a,^ **	−0.20 ^a,^ **
(IC 95%)	(−0.38, −0.15)	(−0.33, −0.09)
Number of sons/daughters	−0.03 ^a^	−0.02 ^a^
(IC 95%)	(−0.16, 0.10)	(−0.14, 0.10)
Responsibility for the care of family members (IC 95%)	0.13 ^b^	0.10 ^b^
	(−0.02, 0.29)	(−0.05, 0.24)
Lab domest	0.00 ^c^	0.01 ^c^
(IC 95%)	(0.00, 0.00)	(0.00, 0.04)
Homework children	−0.13 ^a^	−0.09 ^a^
(IC 95%)	(−0.26, 0.00)	(−0.23, 0.02)
Years of teaching experience	−0.20 ^a,^ **	−0.17 ^a,^ **
(IC 95%)	(−0.31, −0.08)	(−0.30, −0.04)
Job satisfaction	−0.34 ^a,^ **	−0.23 ^a,^ **
(IC 95%)	(−0.45, −0.21)	(−0.36, −0.11)

Note: WFC is the work–family conflict score, FWC is the family–work conflict score. Maximum likelihood scores (sML) were used for CTF and CFT. ^a^ Spearman correlation. ^b^ Biserial rank correlation. ^c^ Epsilon squared effect size. CI 95%: 1000 simple bootstraps. * *p* < 0.05; ** *p* < 0.01.

## Data Availability

Following the policies of information transparency, the authors support open science and, therefore, make the database accessible to those who request it.
